# Analysis of Fitness Status Variations of Under-16 Soccer Players Over a Season and Their Relationships With Maturational Status and Training Load

**DOI:** 10.3389/fphys.2020.597697

**Published:** 2021-02-05

**Authors:** Hadi Nobari, Ana Filipa Silva, Filipe Manuel Clemente, Marefat Siahkouhian, Miguel Ángel García-Gordillo, José Carmelo Adsuar, Jorge Pérez-Gómez

**Affiliations:** ^1^Department of Exercise Physiology, Faculty of Sport Sciences, University of Isfahan, Isfahan, Iran; ^2^Department of Sport Sciences, University of Mohaghegh Ardabili, Ardabil, Iran; ^3^HEME Research Group, Faculty of Sport Sciences, University of Extremadura, Cáceres, Spain; ^4^Escola Superior Desporto e Lazer, Instituto Politécnico de Viana do Castelo, Rua Escola Industrial e Comercial de Nun’Álvares, Viana do Castelo, Portugal; ^5^N2i, Polytechnic Institute of Maia, Maia, Portugal; ^6^The Research Centre in Sports Sciences, Health Sciences and Human Development (CIDESD), Vila Real, Portugal; ^6^Facultad de Administración y Negocios, Universidad Autónoma de Chile, Sede Talca, Chile

**Keywords:** internal load, monitoring, performance, football, youth

## Abstract

The purposes of this study were (i) to analyze the variations in maximal oxygen consumption (VO_2m__ax_), maximal heart rate (HR_max_), heart rate at rest, acceleration, maximal speed, agility, anaerobic sprint test (RAST) of peak power (RPP), RAST of minimum power, RAST of average power (RAP), and RAST of fatigue index (RFI) during the competitive season, using maturation status and accumulated training load as covariates, and (ii) to describe the differences between responders and non-responders in relation to baseline levels. Twenty-three elite players from the same team competing in the national under-16 competitions were evaluated for 20 weeks in period 1 (before league), middle (mid league), and period 2 (after league). The VO_2m__ax_ (*p* = 0.009), maximal speed (*p* = 0.001), RPP (*p* < 0.001), RAP (*p* < 0.001), and RFI (*p* < 0.001) significantly changed across the assessment periods. Interestingly, using accumulated training load and maturation status as covariates revealed no statistical significance (*p* > 0.05). When analyzing responders and non-responders, only HR_max_ (between periods 1 and 2) showed no differences between the groups. As a conclusion, it can be seen that accumulated training load and maturation status play an important role in the differences observed across the season. Thus, coaches should consider the importance of these two factors to carefully interpret fitness changes in their players and possibly adjust training decisions according to the maturation level of the players.

## Introduction

Successful soccer performance depends on multiple, complex, and interdependent factors, including anthropometric traits, maximal speed, change-of-direction ability (COD), and aerobic and anaerobic capacities (Stolen et al., 2005; Hulse et al., 2012). In fact, soccer is an intermittent high-intensity sport, which includes various types of running with rapid changes of directions, starts, stops, jumps, and kicks (Alfredson et al., 1996). Therefore, physical performance is commonly assessed and monitored as the outcome of standardized motor tasks requiring maximal speed, COD ability, balance, flexibility, explosive strength (Malina et al., 2004), local muscular endurance, and static muscular strength, particularly for the purpose of long-term athletic training (Carling, 2013).

The energy demand in soccer players is mainly dependent on aerobic capacity (Stolen et al., 2005). Adults soccer players usually cover between ∼10 and 12 km, while in young soccer players the distances covered during competitions is lower: 6.5, 7.4, and 8.1 km (for under −13, −14, and −15, respectively) (Buchheit et al., 2010). Moreover, higher levels of aerobic capacity enhance recovery from high-intensity interval loads (Svensson and Drust, 2005). This capacity also seems to be a prerequisite to improve the efficiency of anaerobic capacity when performing high-intensity intermittent efforts (Tomlin and Wenger, 2001). Also, when performing repetitive sprints tests, the best players showed more tolerance to fatigue (Reilly et al., 2000b). Finally, change-of-direction ability is also able to discriminate recreational and non-soccer players matched for intermittent endurance capacity (Coratella et al., 2016).

Maximal speed and COD ability tests are commonly used in talent identification programs (Rommers et al., 2019), as during the match activities involving accelerations (ACC), maximal speed, and COD ability number in the range of 150–250 n (Bangsbo et al., 2007). Although the physiological measures (e.g., absolute maximal oxygen uptake—VO_2m__ax_) were generally more discriminative than anthropometry (Reilly et al., 2000a), positive correlations between body mass, fat-free mass, and skeletal muscle mass have been observed (Boraczyński et al., 2015). Indeed, fat-free mass has been shown to be a significant predictor of maximal speed, endurance, and jump capacity (Gil et al., 2014). Nevertheless, improvements in performance are also dependent on increases in anthropometric traits, body mass, skeletal muscle mass, heart and lung mass, hemoglobin level, and blood volume as well as maturation of the nervous system (Stolen et al., 2005). This highlights the importance/influence of growth and maturity in performance. In fact, the optimal period (window of opportunity) to improve physical, technical, and physiological capacities has been postulated to occur between the ages of 12 and 16 years, during maturation (Lloyd and Oliver, 2012).

During adolescence, the interaction between genes, hormones, nutrients, and environmental factors triggers a series of physical and functional alterations in the body (Borges et al., 2018). The capacities mentioned above show their greatest improvement during the adolescent growth spurt (Pearson et al., 2006). This period does not occur at the same time in all players, favoring the early maturing players. Teixeira et al. (2014) showed that early maturing players are taller and heavier and attained higher absolute values in ventilatory thresholds and maximal peak of oxygen consumption compared to average maturing players, even after normalizing for interindividual variability in anthropometric traits. Because of their advanced anthropometric and physical profile (Figueiredo et al., 2010; Meylan et al., 2010), early maturing footballers play regularly and are more often selected for regional and national teams (Meylan et al., 2010). In fact, the literature has confirmed that players that are born in the first months of the year, normally have an advantage as their maturation is more advanced compared to athletes who were born in the third and fourth quarters of the year (Deprez et al., 2012). In a study that analyzed age and maturity in soccer, Rommers et al. (2019), showed significant differences among several successive age categories, highlighting that the pubertal period is a critical time for skill acquisition and development of performance in youth elite soccer players.

Following the Long-Term Athlete Development model, players aged 16–18 are in the “training to compete” phase of development with highly structured training (Ford et al., 2011). Until that phase, players are still developing their metabolic conditioning, endurance strength, power, and the cognitive skills specific to soccer. Therefore, their response to training load and recovery will be evidently different from adults, who train predominantly to maintain and improve physical traits and performance, as they already have more developed physical characteristics (Lloyd and Oliver, 2012). Thus, to ascertain appropriate loads for each player also regarding their age, it is important to monitor training loads individually (Vahia et al., 2019). In fact, monitoring the training load in soccer is a key component of the training process as it helps set an adequate balance between training and recovery (Gaudino et al., 2015).

The study of youth soccer players’ fitness variations is recurrent in the literature (Vänttinen et al., 2011; Emmonds et al., 2020). However, few studies (Wrigley et al., 2014; Ramirez-Campillo et al., 2019) are found using maturation status or accumulated training load as covariables that interact with the changes in fitness across the season. This factor should be considered since maturation and accumulated training load seem to play an important role in players’ development (Malina, 2014). Additionally, little attention is given to those who improve or do not improve their fitness status across the season. In this respect, an analysis of responders and non-responders is relevant (Atkinson et al., 2019), namely, to identify how the player is responding or not to the process. Eventually, comparing both types of responses may provide additional information to understand the development of players across the season.

Based on the abovementioned reasons, the purposes of this study were (i) to analyze the variations in physiological variables (e.g., VO_2m__ax_, maximal heart rate—HR_max_, heart rate rest—HR_rest_), neuromuscular variables (e.g., ACC, maximal speed, COD ability), and running-based anaerobic sprint capacity (e.g., peak power—RPP, minimum power—RMP, average power (Schrapf and Tilp, 2013), and Fatigue Index—RFI) during a season using maturation status (MS) and accumulated training load (ATL) as covariables and (ii) to analyze the differences between responders and non-responders (players that improved and those that decreased performance) in relation to each time point with the previous step.

## Materials and Methods

### Participants

The participants were 23 male youth elite soccer players (Mean ± Standard deviation; age: 15.45 ± 0.24 years; height: 172.70 ± 4.24 cm; body mass: 61.30 ± 5.62 kg; body fat: 8.34 ± 2.89%; VO_2m__ax_, 48.38 ± 2.55 ml kg^–1^ min^–1^) from the same team competing in the national under-16 competition. From the 23 players, 9 were defenders, 6 were midfielders, 4 were wingers, and 4 were forward. The inclusion criteria were as follows: (i) all players participated in at least 90% of the training season; (ii) no use of dietary supplements was allowed; (ii) no non-contact injuries in the 20-weeks period; (iii) players could not participate in any other training during the study; and (iv) players who did not participate in the match during the week had a separate training session without the ball or practice in a small-sided game. Exclusion criteria are as follows: (i) goalkeepers were excluded based on the high variations in physical demands compared to outfield players. Before starting the study, participants were given explanations about the various stages of the research. All players, along with their parents, were notified of the potential risks and benefits of participating in the study. Players, as well as their parents, signed their informed consent to participate in the project. Prior to the start of the study, the Ethics Committee of the University of Isfahan and the University of Mohaghegh Ardabili approved the study, and the recommendations of Human Ethics in Research were followed according to the Helsinki Declaration.

### Experimental Approach to the Problem

The present study consists of two parts: (i) The first one was a quasi-experimental design with pre-, mid-, and post-tests. In this study, the assessments were performed three times: period 1 (P1 = before league), period 2 (P2 = after league), and middle period (Mid = mid league), in the eleventh week of the study. (ii) The second study was a cohort study with daily monitoring over 20 consecutive weeks in the competition season. Details can be seen in Table 1. This study started in August 2019 and ended in February 2020. The players performed a total of four training sessions, one match with two rest days per week. In each period, the players were assessed on four consecutive days. On the first day, assessments were made of anthropometric and body composition (e.g., height, sitting height, body mass, body fat), MS for calculating peak height velocity (PHV), and maturity offset performed only in P1. On the second day, the maximal speed, ACC, and COD ability with device Newtest Powertimer 300-series testing system made in Finland were measured. On the third day, the anaerobic power was assessed. Finally, on the fourth day, the aerobic power test with a heart rate sensor (Mi-Band 3; Xiaomi Company, China) for maximum heart rate (HR_max_) was conducted. Players were divided into two groups according to the average scores in the P1 assessments: (i) responders, who were above average, and (ii) non-responders, who were below average in each variable (i.e., physiological, neuromuscular, and anaerobic), then comparisons of P1 to the Mid and P2 were performed. Testing sessions were carried out for each participant under similar environmental conditions (21–23°C temperature and 50% humidity) and at the same time on the 3 days of physical fitness tests (Pescatello et al., 2014). In all sessions, the tests were performed between at 3:30 and 6:30 pm. All players reported the training load 30 min after each training session, then each training load was calculated with the training time. Finally, in this study, to evaluate the factors affecting the bio-motor ability of the youth players, ATL and MS (age at PHV) were considered as covariates.

**TABLE 1:** Training, match, and assessed sessions during the period.

Variables	P1	W (1–10)	Mid (W11)	W (12–20)	P2	Total
Weeks (*n*)	1	10	1	9	1	22
Training sessions (*n*)	5	37	4	36	4	86
Matches (*n*)	–	10	–	9	–	19

*P1, period 1 = before league; W, weeks; Mid, middle = mid league; P2, period 2 = after league.*

### Anthropometric and Body Composition

Height (standing and sitting) and weight measurements, respectively, were made with a SECA Model 213 stadiometer, made in Germany with a typical error of 0.5 mm and a SECA scale, model 813, made in England, with a typical error of ± 0.1 kg. Measurements were performed according to the International Society for the Advancement of Kinanthropometry Standards. The Mirwald formula was used to determine maturity offset and age at PHV (Mirwald et al., 2002).

All measurements were performed in the morning by an exercise physiologist with 5 years’ experience (Arazi et al., 2015). The Jackson and Pollock seven-point method (Jackson and Pollock, 1978) was used to determine the body fat percentage with a calibrated Lafayette Skinfold Calliper (Lafayette, IN, United States) with an accuracy of 0.1 mm. All measurements were performed on the right side of the body (chest, abdominal, thigh, triceps, subscapular, suprailiac, and midaxillary). At this point, considerations made in the previous study were used to reduce the measurement error (Nobari et al., 2020). Measurements were taken three times and the average of the three was used for the formula (Nobari et al., 2020).

### Monitoring Internal Training Loads

**FIGURE 1:**
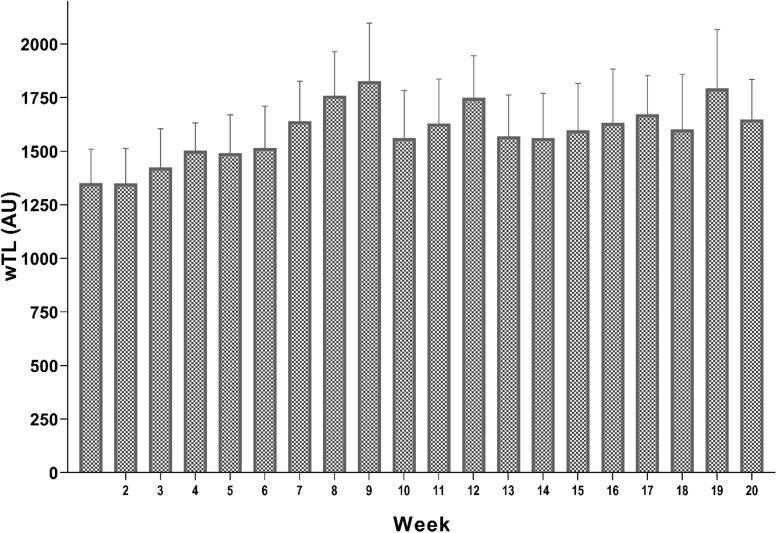
Demonstrated weekly training loads carried out during the 20 consecutive weeks. wTL, weekly training load; AU, arbitrary unit.

Players recorded their rating of perceived exertion (RPE) using the CR-10 Borg’s scale (Borg, 1998) that is a valid and reliable scale to estimate the intensity of a session. Thirty minutes after the end of the training session, the RPE of each player was noted between 0 (minimum effort) and 10 (maximal effort) after answering this question “What was the intensity of your session?” As a measure of internal load, the session-RPE was calculated multiplying the score on the CR-10 scale by the duration of the session in minutes (Foster et al., 2001). Players were familiar with the scale and had used it for 2 years in the club. Total ATL for training and competition was calculated as follows: the first weekly TL+ the second weekly TL+…+ up to the twentieth week. Figure 1 presents information about the general characteristics of the weekly training loads carried out during the 20 consecutive weeks in the competitive season.

### Aerobic Power Test

The Intermittent Fitness Test 30-15 (30-15_I__FT_) was taken to calculate the VO_2m__ax_ and the readiness level of the subjects (Buchheit, 2008, 2010). This test consists of 30 s shuttle runs interspaced with 15 s passive recovery periods. The initial velocity was set at 8 km h^–1^ with 0.5 km h^–1^ increments for each stage. After 10 min of a standard warm-up, subjects were divided into groups of four and stood on the start line (A); when they heard the first beep, they were required to run back and forth between 2 lines set 40 m apart at a pace marked by a prerecorded beep. During the 15 s recovery time, subjects walked forward to the closest line depending on where they had stopped the previous run and began the next run from this line. This test went on until subjects could no longer continue the test, or three consecutive times could not reach the two end lines. The 30-15_I__FT_ was used to determine VO_2m__ax_ with the following formula (Buchheit, 2010): VO_2m__ax_ (ml kg^–1^ min^–1^) = 28.3 – (2.15 × 1) – (0.741 × 16 years) – (0.0357 × weight) + (0.0586 × 16 years × VIFT) + (1.03 × VIFT). VIFT was the final running speed attained. The test–retest reliability of the test was calculated with an intra-class correlation coefficient (ICC). The ICC was 0.86 for this test.

### Heart Rate Maximum and Resting Measurement

The HRmax calculation test was performed with the Mi-Band 3 in the 30-15_I__F__T_ test. Each subject has been monitoring with the watch on the wrist. HR_max_ was recorded with the Mi-Band 3 in the 30-15_I__F__T_ test, and each subject was fitted with the monitor on his wrist before each test. HR_max_ recorded during the test was considered as a reporting criterion of HR_max_. To measure resting heart rate (HRrest), the recommendation was to measure their HRrest in the morning after a rest day. The measurement had to be performed in a supine position in the morning immediately after waking up. Subjects put an HR sensor (Mi-Band 3; Xiaomi Company, made in China) on their wrist, lay down on their backs, and stayed in a relaxed position. After about 10 min, they could start the HR monitor. They continued to lie still and breathe calmly for 3–5 min without looking at the monitor. Then they stopped it. This process was repeated for 3 days, then the researcher checked the summary for their average HR for the 3 days, which was taken as HRrest for all players.

### Acceleration and Maximal Speed Test

For the acceleration and maximal speed test, a digital timer connected to two photocells was placed at hip height, and after a 10 min specific warm-up subjects stood 70 cm behind the start line (line “A”). To calculate the ACC, when the voice from the speakers said: Ready, Go! subjects started to run from the start line, where the first photocell gate was placed, to the 10 m as fast as possible. When subjects passed through the first photocell gate, the digital timer started, and when they reached the second gate the timer stopped, recording the ACC for each subject.

To calculate maximal speed (Mirkov et al., 2008), the test was performed over a distance of 30 m. The best value obtained from 3 trials was used for statistical analysis. They had at least 3 min rest between each trial, and all phases of testing were monitored by the coach. The ICC was 0.91 for ACC and 0.94 for maximal speed.

### Change-of-Direction Test

The soccer players did a “modified 505” (Sheppard and Young, 2006) after the warm-up, they rested for 3–5 min to recover. Then, subjects stood 70 cm behind the start line “A.” Line “B” and line “C” were placed 5 and 10 m, respectively, from “A.” The tests were timed by photocells placed on line “B.” All the participants did the test twice with a 3 min recovery in between. They had to touch line C with their hands and then change direction as quickly as possible and return to the start line “A.” The tests were supervised by the coach or researcher, and the Photo-finish system recorded the time taken to complete the 5 m (out and return) (2 × 5 m). The best time was used for the statistical analysis. The ICC for the COD was equal to 0.94.

### Anaerobic Test

A RAST was used to measure anaerobic power. To perform this test, two photocells were placed at 35 m from each other. Each participant had to run at maximal speed for 6 repetitions and take a 10 s rest between each repetition. After performing a warm-up following the same instructions as for the other tests, the participants began running at maximal speed. After crossing the 35 m line, they rested for 10 s (seconds were counted aloud by the investigator) and then immediately started again at the end of 10 s; this process continued for six repetitions. The records of each participant were calculated with the following formulas and considered for anaerobic power variables; RAST of peak power (RPP) = the highest value; RAST of minimum power (RMP) = the lowest value; RAST of average power (Schrapf and Tilp, 2013) = sum of all six values ÷ 6; and RAST of Fatigue Index (RFI) = (Maximum power − Minimum power) ÷ Total time for the 6 sprints. Test–retest reliability calculated with the ICC was 0.91 for this test. All maximal speed, COD, and anaerobic power tests were performed using the Newtest Powertimer 300-series testing system made in Finland.

### Statistics

Homogeneity of the data was verified with the Levene test and the normality of the data with the Shapiro–Wilk test. Data (i.e., aerobic and anaerobic power, HR measurements, and speed variables) were analyzed using repeated-measure ANOVA with covariates (accumulated training load and PHV). In the model, they show the following values: accumulated training load = 32082.44 arbitrary units (AU) and age at PHV = 13.60 years. Then, all variables were analyzed with the LSD *post hoc* test. Hedge’s g effect size model was calculated for different time points regarding pairwise comparisons. The interpretation of magnitude of changes was as follows (Batterham and Hopkins, 2006; Hopkins et al., 2009): trivial < 0.2; small = 0.01; moderate = 0.6–1.2; large = 1.2–2.0; very large = 2.0–4.0; and extremely large≥4.0. Finally, the independent *t*-test divided responders and non-responders by the cutoff values considering the average scores in P1 assessments for each variable and then comparing P1 to the Mid and P2 (comparison of groups from the P1 is done, and this way the groups are considered the same in all comparisons.) We calculated the ICC for reliability (including neuromuscular variables) and test–retest reliability (including aerobic and anaerobic variables). Data analysis was performed using SPSS software (version 23.0), and the significance level was set at *P* < 0.05. We performed to calculate an a posteriori estimation of statistical power; the statistical software (G-Power; University of Dusseldorf, Dusseldorf, Germany) was used. Given the study design (the difference between two independent means for two groups), accordingly, an alpha risk of 0.05 was used in a two-sided test.

## Results

The individual characteristics of the subjects are presented in Table 2.

**TABLE 2:** Descriptive characteristics of U16 soccer players (*n* = 23) by playing positions.

Playing position	DF (*n* = 9)		Mid (*n* = 6)		WG (*n* = 4)		FW (*n* = 4)	
Characteristic	Mean	*SD*	Mean	*SD*	Mean	SD	Mean	*SD*
Age (years)	15.5	0.2	15.4	0.2	15.3	0.4	15.6	0.1
Height (cm)	175.1	4.5	171.60	2/1	170.5	2.9	172.8	5.3
Weight (kg)	65.1	5.4	58.4	2.1	56.9	4.8	61.4	6.1
Experience (years)	6.2	1.6	6.7	1.8	5.3	1.3	6.5	1.9
Sitting height (cm)	93.4	2.5	92.2	1.2	91.4	2.2	92.6	1.8
Body Fat (%)	12.4	3.1	9.5	2.6	9.28	2.5	11.8	2.25
ATL (AU)	31810.9	812.1	32562.0	1683.7	32855.3	990.1	31201.3	967.6
**Maturation (years)**								
PHV	13.5	0.4	13.7	0.2	13.8	0.4	13.6	0.3
Maturity offset	2.0	0.3	1.7	0.2	1.6	0.2	1.9	0.2

*SD, standard deviation; Age at PHV, number of years the athlete is away from peak height velocity; DF, defenders; Mid, midfielders; WG, wingers; FW, forward; ATL, accumulated training load; AU, arbitrary unit.*

The repeated-measure ANOVA of the VO_2m__ax_ demonstrated main effects of time [*F*(2, 5.20), *P* = 0.009], whereas after the entry of the ATL [*F*(1, 0.95), *P* = 0.343] and MS [*F*(1, 1.39), *P* = 0.253] as covariates, there was not a statistical significance between time points (Table 3). *Post hoc* tests showed that there was a significant difference between P1-mid (*P* = 0.001) as well as with a covariate ATL and MS (*P* = 0.001). The overall effect size was small (g = 0.34) (Table 4).

**TABLE 3:** Accumulated training load and maturation status data for fitness tests and relationship with different time points.

Variables	P1 Mean ± SD	Mid Mean ± SD	P2 Mean ± SD	Main effect of Time		ATL × Time Effect		MS × Time Effect	
				*F*	*P*	*F*	*P*	*F*	*P*
VO_2m__ax_ (ml⋅kg^–1^⋅min^–1^)	48.38 ± 2.55	49.28 ± 2.69	48.74 ± 2	5.20	0.009*	0.95	0.343	1.39	0.253
HR_max_ (bpm)	194.74 ± 4.37	193.83 ± 5.01	193.65 ± 5.4	0.50	0.515	0.75	0.417	0.72	0.425
HR_rest_ (bpm)	54.61 ± 4.21	54.83 ± 4.24	55.35 ± 4.03	3	0.090	1.99	0.171	0.692	0.439
ACC (s)	1.80 ± 0.24	1.83 ± 0.27	1.83 ± 0.26	2.13	0.152	0.21	0.698	0.98	0.351
Max speed (s)	4.20 ± 0.32	4.17 ± 0.34	4.08 ± 0.32	11.43	0.001*	0.59	0.513	0.06	0.894
AG (s)	2.36 ± 0.17	2.40 ± 0.19	2.40 ± 0.19	0.920	0.374	2.12	0.155	0.12	0.790
RPP (W)	734.96 ± 156.4	741.29 ± 152.9	837.75 ± 168.5	30.96	< 0.001*	0.99	0.335	0.57	0.46
RMP (W)	407.91 ± 98.3	412.73 ± 97.9	433.73 ± 114.6	2.55	0.124	1.54	0.230	0.69	0.418
RAP (W)	553.41 ± 115.3	559.19 ± 115.9	614.01 ± 134.4	24.85	< 0.001*	0.009	0.927	0.001	0.983
RFI (W/s)	10.56 ± 3.7	10.80 ± 3.4	13.98 ± 4.1	34.46	< 0.001*	0.28	0.666	2.56	0.114

P1, period 1; Mid, middle; P2, period 2; SD, standard deviation; ATL, accumulated training load; MS, maturation status; VO_2m__ax_, maximal oxygen consumption; HR_max_, maximal heart rate; HR_rest_, resting heart rate; ACC, acceleration; Max speed, maximal speed; AG, agility; RPP, RAST of peak power; RMP, RAST of minimum power; RAP, RAST of average power; RFI, RAST of Fatigue Index; bpm, beats per minutes; W, watts; S, seconds. *Significance of main effect in time at the 0.05 level.

**TABLE 4:** Accumulated training load and maturation status data for fitness tests in relation to different time points and pairwise comparisons.

Variable	Time point	MD	95% CI for D		LSD	ATL and MS	Hedge’s g
			Lower	Upper	*p*	*p*	Value (magnitude)
VO_2m__ax_ (ml⋅kg^–1^⋅min^–1^)	P1-Mid	−0.903*	–1.37	–0.44	0.001^#^	< 0.001^∞^	0.34*S*
	Mid-P2	0.540	–0.03	1.11	0.061	0.068	−0.22*S*
	P1-P2	–0.362	–1.06	0.33	0.292	0.298	0.15*T*
HR_max_ (bpm)	P1-Mid	0.913	–0.11	1.93	0.076	0.067	−0.19*T*
	Mid-P2	0.174	–2.81	3.16	0.905	0.905	−0.03*T*
	P1-P2	1.087	–1.67	3.85	0.423	0.434	−0.22*S*
HR_rest_ (bpm)	P1-Mid	–0.217	–0.48	0.04	0.096	0.110	0.05*T*
	Mid-P2	–0.522	–1.29	0.25	0.174	0.169	0.12*T*
	P1-P2	–0.739	–1.50	0.02	0.057	0.056	0.17*T*
ACC (s)	P1-Mid	–0.032	–0.07	0.01	0.106	0.109	0.12*T*
	Mid-P2	0.002	–0.08	0.02	0.812	0.804	−0.01*S*
	P1-P2	–0.030	–0.07	0.01	0.178	0.198	0.12*T*
	P1-Mid	0.026	–0.01	0.07	0.191	0.204	−0.08*T*
Max speed (s)	Mid-P2	0.099*	0.04	0.16	0.002^#^	0.003^∞^	−0.29*S*
	P1-P2	0.125*	0.06	0.19	0.001^#^	0.002^∞^	−0.38*S*
AG (s)	P1-Mid	–0.016	–0.04	0.01	0.221	0.180	0.08*T*
	Mid-P2	–0.002	–0.03	0.02	0.842	0.845	0.01*S*
	P1-P2	–0.018	–0.06	0.02	0.348	0.360	0.10*T*
RPP (W)	P1-Mid	−6.330*	–11.03	–1.63	0.011^#^	0.014^∞^	0.04*T*
	Mid-P2	−96.465*	–133.62	–59.31	< 0.001^#^	< 0.001^∞^	0.58*M*
	P1-P2	−102.796*	–139.73	–65.86	< 0.001^#^	< 0.001^∞^	0.62*M*
RMP (W)	P1-Mid	−4.817*	–7.74	–1.89	0.002^#^	0.004^∞^	0.05*T*
	Mid-P2	–21.009	–51.65	9.63	0.169	0.166	0.19*S*
	P1-P2	–25.826	–56.82	5.17	0.098	0.096	0.24*S*
RAP (W)	P1-Mid	−5.783*	–8.69	–2.87	< 0.001^#^	0.001^∞^	0.05*T*
	Mid-P2	−54.822*	–78.57	–31.08	< 0.001^#^	< 0.001^∞^	0.43*S*
	P1-P2	−60.604*	–84.88	–36.33	< 0.001^#^	< 0.001^∞^	0.47*S*
RFI (W/s)	P1-Mid	–0.242	–0.72	0.24	0.308	0.315	0.07*T*
	Mid-P2	−3.184*	–4.30	–2.07	< 0.001^#^	< 0.001^∞^	0.82*M*
	P1-P2	−3.426*	–4.56	–2.30	< 0.001^#^	< 0.001^∞^	0.86*M*

P1, period 1; Mid, middle; P2, period 2; MD, mean difference; D, difference; CI, confidence interval; ATL, accumulated training load; MS, maturation status; VO_2m__ax_, maximal oxygen consumption; HR_max_, maximal heart rate; HR_rest_, resting heart rate; ACC, acceleration; Max speed, maximal speed; AG, agility; RPP, RAST of peak power; RMP, RAST of minimum power; RAP, RAST of average power; RFI, RAST of Fatigue Index; bpm, beats per minute; W, watts; s, seconds; T, trivial; S, small; M, moderate; VL, very large; L, large; EL, extremely large. *The mean difference is significant at the 0.05 levels. ∞Represents a statistically significant change in pairwise comparisons LSD at the 0.05 level. ^#^Represents a statistically significant change in pairwise comparisons LSD with ATL and MS at the 0.05 level.

The RPP demonstrated main effects of time [*F*(1.02, 30.96), *P* ≤ 0.001] while, after the entry of the ATL [*F*(1.02, 0.99) = 0.34] and MS [*F*(1.03, 0.57), *P* = 0.46] as a covariate, no significant difference between time points was noticed (Table 3). *Post hoc* tests showed there was a significant difference between P1-mid (LSD; *P* = 0.011 and with a covariate ATL and MS; *P* = 0.014; *g* = 0.04, trivial), mid-P2, and P1-P2 (*P* ≤ 0.001) with and without considering the covariate. In both these time periods, effect size was moderate (Table 4).

The RFI demonstrated main effects of time [*F*(1.28, 34.46), *P* ≤ 0.001] while, after the entry of the ATL [*F*(1.30, 0.28), *P* = 0.666] and MS [*F*(1.30, 2.56), *P* = 0.114] as a covariate, there was not a significant result between time points (Table 3). *Post hoc* tests showed that there was a significant difference between mid-P2 (*P* ≤ 0.001; *g* = 0.82 moderate) and P1–P2 (*P* ≤ 0.001; *g* = 0.86 moderate) with and without considering the covariate.

*Post hoc* power analysis was performed to identify the power obtained for *t*-test analysis, based on an alpha risk of 0.05 in a two-sided test. Statistical power acceptance was for VO_2m__ax_ (97%), HRrest (99%), Max speed (99%), AG (94%), RPP (99%), RAP (98%), and RFI (96%). Therefore, a statistically significant difference can be identified.

Table 5 presents the differences in performance between responders and non-responders. Between P1 to mid and P1 to P2, all variables showed significant results (all with *P* < 0.001) between responders and non-responders. Only HR_max_ showed no differences between responders and non-responders at P1 to P2.

**TABLE 5:** Between-group differences of physiological variables (mean ± standard deviation) between responders and non-responders considering the baseline levels.

Variables	Time point	Responder	Non-responder	Mean difference		P	Hedge’s g Value (magnitude)
				Value	[95% CI]		
VO_2m__ax_ (ml kg^–1^ min^–1^)	P1-Mid	51.79 ± 1.87	47.67 ± 1.71	4.12	[2.55; 5.69]	< 0.001*	2.24*VL*
	P1-P2	50.34 ± 1.89	47.71 ± 1.29	2.63	[1.26; 4.00]	0.001*	1.64*L*
HRmax (bpm)	P1-Mid	197.82 ± 3.46	190.17 ± 2.95	7.65	[4.87; 10.43]	< 0.001*	2.30*VL*
	P1-P2	193.75 ± 6.06	193.55 ± 4.87	0.20	[−4.60; 5.00]	0.930	0.04*T*
HRrest (bpm)	P1-Mid	51.10 ± 2.42	57.69 ± 2.84	–6.59	[−8.93; −4.26]	< 0.001*	−2.38*VL*
	P1-P2	52.10 ± 2.92	57.85 ± 2.79	–5.75	[−8.24; −3.25]	< 0.001*	−1.95*VL*
ACC (s)	P1-Mid	1.58 ± 0.06	2.07 ± 0.14	–0.49	[−0.58; −0.39]	< 0.001*	−4.31*EL*
	P1-P2	1.59 ± 0.05	2.05 ± 0.13	–0.47	[−0.56; −0.38]	< 0.001*	−4.42*EL*
Max speed (s)	P1-Mid	3.92 ± 0.20	4.45 ± 0.23	–0.54	[−0.72; −0.35]	< 0.001*	−2.38*VL*
	P1-P2	3.87 ± 0.23	4.30 ± 0.25	–0.43	[−0.64; −0.22]	< 0.001*	−1.73*L*
AG (s)	P1-Mid	2.26 ± 0.79	2.51 ± 0.19	–0.25	[−0.72; 0.22]	< 0.001*	−0.45*S*
	P1-P2	2.28 ± 0.10	2.50 ± 0.19	–0.22	[−0.36; −0.08]	0.004*	−1.37*L*
RPP (W)	P1-Mid	873.33 ± 57.75	597.24 ± 63.73	276.08	[223.41; 328.75]	< 0.001*	4.39*EL*
	P1-P2	968.18 ± 105.13	695.47 ± 87.32	272.70	[188.45; 356.96]	< 0.001*	2.71*VL*
RMP (W)	P1-Mid	498.45 ± 44.93	334.14 ± 56.86	21.51	[119.59; 209.04]	< 0.001*	3.07*VL*
	P1-P2	516.99 ± 357.42	357.42 ± 44.70	34.44	[87.94; 231.20]	< 0.001*	0.62*M*
RAP (W)	P1-Mid	647.81 ± 50.93	443.99 ± 57.70	203.82	[156.64; 250.99]	< 0.001*	3.64*VL*
	P1-P2	704.84 ± 88.69	495.94 ± 79.18	208.90	[134.77; 283.03]	< 0.001*	2.37*VL*
RFI (W/s)	P1-Mid	13.98 ± 2.18	8.36 ± 1.78	5.61	[3.90; 7.33]	< 0.001*	2.76*VL*
	P1-P2	16.84 ± 4.33	11.79 ± 2.12	5.05	[2.20; 7.90]	0.001*	1.50*L*

P1, period 1; mid, middle; P2, period 2; VO_2m__ax_, maximal oxygen consumption; HR_max_, maximal heart rate; HR_rest_, resting heart rate; ACC, acceleration; Max speed, maximal speed; AG, agility; RPP, RAST of peak power; RMP, RAST of minimum power; RAP, RAST of average power; RFI, RAST of Fatigue Index; bpm, beats per minute; W, watts; s, seconds; T, trivial; S, small; M, moderate; VL, very large; L, large; EL, extremely large. *Represents a statistically significant between group from baseline at the 0.05.

## Discussion

This study aimed to analyze the variations in physiological, neuromuscular, and running-based anaerobic sprint variables during a season using maturation status and accumulated training load as covariables. Additionally, it was also the purpose of this study analyze the differences between responders and non-responders (players that improved and those that decreased performance) in relation to each time point with the previous step. The main evidence of the current study revealed that VO_2m__ax_, maximal speed, RPP, RAP, and RFI significantly changed across the assessment periods. Interestingly, using accumulated training load and maturation status as covariates revealed no statistical significance. When analyzing responders and non-responders, only HR_max_ (between periods 1 and 2) showed no differences between the groups.

In the first period of evaluation, increases in VO_2m__ax_, RPP, and RAP were noticed with and without the inclusion of the ATL and MS covariates. In fact, soccer requires a high aerobic capacity, as the duration of a match is 90 min, and although less abundant compared with adults, studies on youth soccer report distances of 6.5 km (Under-13), 7.4 km (Under-14), and 8.1 km (Under-15) covered during competitions (Stolen et al., 2005). The focus of its increment seems to be in the preseason (about 27.2% in endurance performance) (Dragijsky et al., 2017), which could explain only a small effect in the results of the present study, as the analysis was initiated already at the beginning of the league competitions, excluding the preseason. Nevertheless, in other studies (Bangsbo, 1994; Haritonidis et al., 2004), an increased aerobic endurance level was reported at the beginning to the middle of the season. Likewise, repeated high-speed actions are considered a crucial physical component in soccer matches (Buchheit et al., 2010), as during a match some 150–250 are performed (Bangsbo et al., 2007). However, studies in young soccer players seem to suggest that its stimulation does not occur during the preseason (Dragijsky et al., 2017), as opposed to adults (Dianzenza et al., 2009). However, this study does not cover the pre-season period. However, in the first period of competition, a seemingly good progress was observed in the RAP and RPP variables with trivial and moderate effect size, respectively.

From the mid to the P2 periods, maximal speed, RPP, RAP, and RFI increased. In fact, considering the anaerobic variables related to sprinting, only RMP did not record significant results. During a soccer game, sprinting bouts generally occur every 90 s and each of these sprints last approximately 2–4 s (Bangsbo et al., 2006; Burgess et al., 2006), totaling 1,000–1,400 high-intensity short-duration activities (Stolen et al., 2005). Thus, preserving a high level of these capacities throughout the season is necessary for achieving consistent high-quality performance (Reilly et al., 2000a), while the basis for these individual players’ components are built during youth (Dragijsky et al., 2017). The literature has shown that elite players are faster than sub-elite players in maximal speed tests, and sprint time over 15 m was the strongest discriminator no matter the player’s position. Hence, elite players were found to better reproduce their top speed in the repetitive sprint tests and were more tolerant to fatigue (Reilly et al., 2000b). The results of the present study seem to follow this assumption, as a moderate effect size was recorded for RPP and RFI between the mid and P2 periods, confirming the focus of their coach to develop sprint capacity. Likewise, although with a lower effect (small), maximal speed and RAP also showed significant improvements in this period.

When analyzing the evolution of soccer matches, a greater focus on sprint abilities rather than aerobic capacity has been observed in different studies, underlying that elite or professional players have become faster over time while aerobic capacity has plateaued or slightly decreased (Haugen et al., 2013). On the one hand, these concerns seem to be confirmed in the present study, since in the second observation period (mid to P2 period), almost all speed components showed significant increases over the 20 weeks of analysis. The effect was also moderate for RPP and RFI, and small for maximal speed and RAP. On the other hand, the player’s age and stage of development, i.e., biological maturation, should be considered as they have a great influence on maximal speed, power, and strength improvements (Dragijsky et al., 2017). This capacity, which is considered a complex fitness trait, is apparently related to neuromuscular (Buchheit, 2012) and metabolic factors (Girard et al., 2011) and is strongly based on the myelination of motor nerves and neural maturation.

This process is not complete until sexual maturity is reached (De Ste Croix et al., 2003). In fact, muscle strength in the lower limbs has been considered to increase up to 50% between 11 and 15 years in boys (Degache et al., 2010), influencing sprint ability that improves progressively from ages 11 to 17 years (Valente-dos-Santos et al., 2012).

During adolescence, the interaction between genes, hormones, nutrients, and environmental factors triggers a series of physical and functional alterations in the body (Borges et al., 2018). The literature has shown that physical performance is highly influenced by maturation (Vandendriessche et al., 2012), it being the optimal period (window of opportunity) to improve physical, technical, and physiological capacities between the ages of 12 and 16 years (Balyi and Hamilton, 2004). In fact, studies have highlighted that early maturating players are at an advantage, as they have shown to have greater height, body mass, strength, and aerobic endurance (Teixeira et al., 2018). Therefore, the pubertal period is a critical time frame for skill acquisition and development of performance in youth elite soccer players (Rommers et al., 2019). All of the above could explain the impact observed with the inclusion of MS and ATL covariates, since the players that participated in the present study are within these ages.

The analysis between responders and non-responders made it possible to confirm that the variation in response around the mean alludes to an interindividual variation in exercise and training responses, since only HR_max_ (between P1 and P2) showed no differences between these groups. Indeed, it is known that HR_max_ is relatively unaltered regardless of training status in a given group (Zavorsky, 2000). The non-responder term has been used to describe individuals who showed a worsened or unchanged response after training (Rampinini et al., 2007) or, more accurately, individuals whose training response did not exceed the variation of a particular measurement (Vigne et al., 2010). In the present study, this comparison strengthened the impact of covariates, i.e., the ATL perception and the MS could really influence the performance improvement, although the average values did not always indicate this. In fact, in all variables where improvements were recorded in the whole group (mean values), both MS and ATL showed their impact.

The current study presents some limitations. One of them is the small sample tested. Eventually, future research should include a larger sample to reinforce the power of the evidence. Additionally, monitoring both internal and external training load during the periods may be interesting to add more information about the discriminating factor of different types of stimuli. Future research should consider also monitoring additional activities such as weight room training or regular physical activity performed extra to field-based training. A final limitation is the fact that responders and non-responders were not specifically analyzed using the smallest worthwhile change. Future studies should use such an approach to better determine if a player can be a bad responder to the training.

Despite these limitations, and as a conclusion, maximal speed and its derived variables seem to be the focus in this age group of soccer players, as significant improvements were noticed in the whole evaluated period, especially between mid and P2. In the first period of evaluation, the main concern of their coach would be the improvement of aerobic capacity, as VO_2m__ax_ showed significant improvements in the whole group. All these variables seemed to be influenced by ATL and MS, since when included as covariates, the differences vanished. Additionally, almost all variables presented differences between responders and non-responders, highlighting the individual responses to training.

As a practical application, the relevance of accumulated training load and maturation status on the development of the fitness level of the players should be emphasized. Thus, coaches should consider adapting the training stimuli to the specific characteristics of the players (namely, maturation status) as well as not interpreting changes in fitness exclusively focusing on the training, but also weighing the maturation process. Possibly, more individualized training is needed, as well as a better relationship between player’s assessment and monitoring and the training plan.

## Data Availability Statement

The original contributions presented in the study are included in the article/supplementary material, further inquiries can be directed to the corresponding authors.

## Ethics Statement

The studies involving human participants were reviewed and approved by the Ethics Committee of the University of Isfahan and the University of Mohaghegh Ardabili. Written informed consent to participate in this study was provided by the participants’ legal guardian/next of kin.

## Author Contributions

HN, FC, and JP-G designed the study and drafted the manuscript. HN performed the experiments. HN, AS, MS, MG-G, and JA participated in the data analysis and drafted the manuscript. JP-G, FC, and HN revised the critical manuscript. All authors read and approved the final version of the manuscript.

## Conflict of Interest

The authors declare that the research was conducted in the absence of any commercial or financial relationships that could be construed as a potential conflict of interest.
